# CircSP3 encodes SP3-461aa to promote ccRCC progression via stabilizing MYH9 and activating the PI3K-Akt signaling pathway

**DOI:** 10.7150/jca.100706

**Published:** 2024-09-16

**Authors:** Xiaoliang Wu, Guoliang Sun, Ruixin Fan, Kai Liu, Chen Duan, Xiongmin Mao, Huahui Wu, Xiangyang Yao, Bo Li, Ke Chen, Yangjun Zhang, Zhong Chen

**Affiliations:** 1 Department of Urology, Tongji Hospital, Tongji Medical College, Huazhong University of Science and Technology, Wuhan, Hubei 430000, China.; 2 Department of Urology, Zhongnan Hospital of Wuhan University, Wuhan, Hubei 430000, China.; 3 Department of Urology, The First Affiliated Hospital, School of Medicine, Zhejiang University, Hangzhou 310000, China.

**Keywords:** Clear cell renal cell carcinoma, circular RNA, metastasis, proliferation, protein coding.

## Abstract

Clear cell renal cell carcinoma (ccRCC) is a primary kidney cancer with high aggressive phenotype and extremely poor prognosis. Accumulating evidence suggests that circular RNAs (circRNAs) play pivotal roles in the occurrence and development of various human cancers. However, the expression, clinical significance and regulatory role of circRNAs in ccRCC remain largely unclear. Here we report that circSP3 to be increased in tissues from ccRCC patients and ccRCC cells, and to positively correlate with ccRCC malignant features. Knockdown of circSP3 inhibits proliferation, triggers apoptosis, and reduces migration and invasion in different ccRCC cells *in vitro*. Correspondingly, circSP3 overexpression Promote ccRCC tumorigenicity in a mouse xenograft model. Mechanistically, circSP3 could bind with the ribosome to initiate the translation process to encodes a novel 461-amino acid peptide referred to as SP3-461aa, which protects the MYH9 protein from proteasomal degradation. SP3-461aa played a pivotal role in mediating the oncogenic effects of circSP3 by interacting with the MYH9 protein and activating the PI3K-Akt signaling pathway. These findings suggested that circSP3 plays an important role in ccRCC development and could be a potential biomarker for the treatment and prognosis of ccRCC.

## Introduction

Clear cell renal cell carcinoma (ccRCC) is the most common type of kidney cancer and is infamous for its high aggressiveness. In spite of surgery being the primary treatment for renal cell carcinoma, more than 30% of patients suffer recurrences and/or metastases after surgery, leading to poor outcomes 1. While there have been substantial improvements in diagnosis and a higher 5-year survival rate, the overall prognosis remains less than ideal 2. Therefore, it is essential to conduct a detailed exploration of the molecular pathways underlying the progression and metastasis of clear cell renal cell carcinoma (ccRCC), with the aim of identifying novel and reliable targets for therapeutic intervention.

The protein methyltransferase SET and MYND domain-containing protein 2 (SMYD2) acts as a histone lysine methyltransferase and has been acknowledged as a potential oncogene in various cancer categories, such as lung adenocarcinoma, gastric cancer 3, 4. SMYD2, a well-regarded lysine methyltransferase, is capable of methylating lysine 4 and 36 (H3K4 and H3K36) residues within histones and non-histone molecules. Additionally, SMYD2 can stimulate the phosphorylation of AKT and ERK1/2 by modulating PTPN13 in breast cancer cells 5. Recent research has proposed that SMYD2 hinders the activity of P53, leading to the promotion of cancer cell progression in several tumor types, including cervical cancer and teratocarcinoma 6, 7. SMYD2-mediated RB1 methylation accelerates cell cycle progression by enhancing RB1 phosphorylation 8. In breast cancer, SMYD2-induced methylation has a suppressive effect on PTEN tumor suppressor function, resulting in the activation of the phosphatidylinositol 3-kinase-AKT pathway 9. In our previous research, SMYD2 was found to promote the progression of renal clear cell carcinoma 10. However, the regulatory mechanism of SMYD2 in the modulation of circRNA remains unclear, this provides a compelling impetus for further exploration of its function in the context of renal clear cell carcinoma.

Multiple circRNAs have been associated with tumor progression 11. However, their functions within the ccRCC remain unclear. It has been established that CircCHST15 is a prognostic marker that modulates the miR-125a-5p/EIF4EBP1 axis in clear cell renal cell carcinomas, promoting cell proliferation and metastasis 12. Circ-TNPO3, a circular RNA, has been identified as a suppressor of metastasis in clear cell renal cell carcinoma by interacting with IGF2BP2 and promoting the degradation of SERPINH1 mRNA 13. It has been found that Circ-AKT3 regulates miR-296p/E-cadherin signaling to suppress metastasis in clear cell renal cell carcinoma 14. In our study, we identified hsa_circ_0002642, also known as circSP3, which showed increased expression levels in ccRCC specimens and was linked to unfavorable clinicopathological features in ccRCC patients. We have determined that circSP3 drives proliferation and metastasis in ccRCC by encoding a 461-amino acid polypeptide. This polypeptide interacts with MYH9, preventing its proteasomal degradation, and activates the PI3K-Akt pathway, thus enhancing circSP3's oncogenic functions. In summary, our study underscores the potential of circSP3 as a novel prognostic marker and therapeutic target for ccRCC 12. Nevertheless, the circRNAs that have been most extensively investigated are predominantly derived from coding exons of genes, and they tend to localize in the cytoplasm 15. Within exonic circRNAs, there may be ORFs that overlap their corresponding mRNAs, or specific ORFs. And a subset of circRNAs previously considered noncoding may possess translational capabilities 16. To illustrate, CircMAP3K translates into a novel protein termed circMAP3K4-455aa, which plays a significant role in driving hepatocellular carcinoma progression and may serve as a promising biomarker 17. In addition to hepatocellular carcinoma, numerous tumor types have been found to contain protein-coding circRNAs, including colorectal cancer and triple-negative breast cancer 18-21. While protein-coding circRNAs are known to be pivotal in tumorigenesis, their involvement in ccRCC and the identification of their functional products remain uncertain.

In this study, we ascertained the upregulation of hsa_circ_0002642 (circSP3) in ccRCC tissues and found a significant correlation with the clinicopathological traits of individuals diagnosed with ccRCC. SMYD2 promotes the expression of circSP3. Cell proliferation, migration, and invasion were suppressed in ccRCC cells by inhibiting circSP3 with small hairpin RNAs (shRNAs). Additionally, we identified a 461-amino acid peptide encoded by circSP3. Through interaction with MYH9 and activation of the PI3K-Akt signaling pathway, SP3-461aa played an important role in mediating the oncogenic effects of circSP3. As a result of our research, the protein-coding circSP3 encodes the SP3-461aa peptide that promotes the proliferation and invasion of ccRCC cells.

## Materials and Methods

### Ethics statement and human tissue samples

The Ethical Committee of Wu Han University granted approval for all experimental procedures. Samples of tissue were taken from Zhong Nan Hospital patients during surgery and promptly preserved at -80 °C until analysis. For the use of these clinical materials in research, all patients have provided written, informed consent.

### Cell culture

The ccRCC cell lines 786-O and OS-RC-2 were purchased from the Cell Bank of Shanghai Institute of Cell Biology (Chinese Academy of Medical Science, Shanghai, China) and authenticated through Short Tandem Repeat (STR) analysis in August 2023. The culture media used for the 786O, 769P and OS-RC-2 cell lines was RPMI-1640 (Invitrogen-Gibco), which was incubated at 37 °C in a humidified atmosphere containing 5% CO2. CAKI-1 cells were cultured in McCoy's 5A (Gibco), while HK2 and HEK293T cells were maintained in DMEM (Gibco). The ACHN and A498 cell lines were cultured in Minimum Essential Medium (MEM) from Gibco. The cell culture media were supplemented with 10% concentration of Fetal Bovine Serum (FBS) from Thermo Fisher Scientific and 1% penicillin-streptomycin from Gibco.

### Establishment of plasmids and stable transfected cells

The target sequences were incorporated into lentiviral vectors, namely pCDH-MSCV-MCS-EF1-copGFP (System Biosciences, USA) and pLC5-ciR (Geneseed Biotech, Guangzhou, China). The plausibility of all plasmids was verified through DNA sequencing. Subsequently, recombinant lentiviral vectors were transfected into HEK293T cells, in conjunction with pMD2.G and psPAX2 plasmids, to generate recombinant lentivirus. The lentivirus was used to infect the target cells, which were then exposed to a 14-day puromycin treatment. After confirming the efficacy of overexpression or depletion plasmids, the surviving cells were utilized for subsequent experiments. SMYD2 knock out cells were constructed by JinKaiRui Biotech (Wuhan, China).

### RNase R and actinomycin D treatment

Actinomycin D (2 mg/mL) was used as a transcription inhibitor, while DMSO (Sig-ma-Aldrich, USA) was used as a negative control. An RNase R reaction was used to determine and authenticate circRNA characteristics. An RNA sample derived from 786O or OS-RC-2 cells was divided into two equal portions, one of which was treated with RNase R and the other served as a control. A quantity of 2 milligrams (mg) of RNA was subjected to incubation with 3 units per milligram (U/mg) of RNase R for a duration of 30 minutes at a temperature of 37 °C. Subsequently, after treatment with RNase R and actinomycin D, quantitative reverse transcription polymerase chain reaction (RT-qPCR) was performed to ascertain the levels of RNA expression for circSP3 and SP3. The mock group employed beta-actin as the reference for computational pur-poses.

### Colony formation, EdU incorporation, and CCK-8 assay

In colony formation assays, approximately 1000 cells were initially introduced into six-well plates and subsequently cultivated for a period of two weeks. The enumeration of cell colonies was conducted subsequent to the application of crystal violet staining. The Cell-Light EdU Apollo488 *In Vitro* Kit (RiboBio, Guangzhou, China) was utilized to stain the nucleus of proliferating cells with red fluorescence in EdU incorporation assays, while all nuclei were stained with blue fluorescent light. In the CCK-8 assay, the cells were subjected to the corresponding kit and then seeded into 96-well microplates at a concentration of 1000 cells per well. The absorbance at 450 nm was measured for each well at different time intervals.

### Cell apoptosis assays

Commence the procedure by extracting cells from culture dishes and subjecting them to a comprehensive rinse using 1X PBS. The washed cells should be suspended in 1X Annexin V binding buffer. Subsequently, the Annexin V-FITC antibody should be introduced into the cell suspension, adhering to the concentration recommended by the manufacturer. The solution should be gently mixed and subsequently incubated in a light-restricted environment for a duration of 15 minutes. Following the incubation period, the Propidium Iodide (PI) staining reagent should be added to the Annexin V binding buffer at the concentration recommended by the manufacturer. The solution should be gently mixed again and the incubation process should be continued in a dark environment for an additional duration of 5 minutes. In order to finalize the procedure, it is necessary to conduct an analysis of the cell suspension utilizing a flow cytometer, while ensuring that the appropriate excitation and detection wavelengths have been established to effectively capture the fluorescence signals emitted by Annexin V-FITC and PI.

### Migration and invasion assays

Approximately 1 × 10^5 786-O cells or 1.5 × 10^5 OS-RC-2 cells were seeded in the upper chambers of 24-well Transwell plates (Corning, USA) using FBS-free medium, while the lower chambers were filled with complete medium supplemented with 10% FBS as a chemoattractant. In the context of the *in vitro* migration assay, cells that successfully migrated through the Transwell filter within a time frame of 12 hours were subsequently preserved and subjected to staining using crystal violet. In the context of the *in vitro* invasion assay, Matrigel-coated Transwell membranes were employed to evaluate the ability of cells to penetrate both the Matrigel membrane and Transwell filter within a 24-hour timeframe. Subsequently, the stained cells were quantified to determine the relative number of cells that successfully accomplished this task, thereby indicating their proficiency in migration or invasion.

### Wound-healing assay

The cells were seeded and grown in RPMI-1640 medium supplemented with 10% FBS in a 24-well culture plate until confluent. Following this, Cell monolayers were incised with sterile 200 mL pipettes, and any dislodged cells were removed using PBS. The cells were cultured in RPMI-1640 medium devoid of serum to arrest cell proliferation. Subsequently, images were captured at specific time points (0 and 24 hours) using a Nikon Eclipse 80i system and NIS-Elements software.

### Fluorescence *in situ* hybridization (FISH)

In accordance with the established protocol, a fluorescence *in situ* hybridization (FISH) assay was conducted. To summarize, the ccRCC cells were cultured and fixed in a confocal dish, followed by the identification of circSP3 localization within these cells through the utilization of the RNA fluorescence *in situ* hybridization (FISH) assay, employing the FISH kit (GenePharma, Shanghai, China), The images were acquired on Leica STELLARIS 5 WLL Confocal Microscope (Germany). The probe sequences are shown in Table S2 of Additional file 1.

### Luciferase reporter assay

The cells were transfected with Luc2-EMCV-IRES-Report vector or Luc2-IRES-Report vector plasmids, which contained either intact or truncated IRES sequences, employing Lipofectamine 2000 (Invitrogen). A Dual-Luciferase Reporter Assay Kit (Promega) was used to quantify luciferase activities 48 hours after treatment. These results are presented as the luminescence ratios between firefly luciferase and Renilla luciferase.

### Co-immunoprecipitation and Liquid Chromatography-Mass Spectrometry (LC-MS)

Cold lysis buffer containing 1% Triton X-100, 50 mM Tris-7.5, 1 mM EDTA, 150 mM NaCl, and protease inhibitors was used to lyse the cells. Subsequently, A supernatant obtained from cell lysates was incubated with the specified antibodies. Following this, the lysates were combined with protein A/G beads. Subsequently, Following the washing of the beads with a cold lysis buffer and the addition of protein loading buffer, western blotting analysis was performed. Liquid Chromatography-Mass Spectrometry (LC-MS) was employed for the qualitative and quantitative analysis of biomarkers.

### CHX-treatment assay

A CHX-treatment assay was conducted, employing CHX (Selleck Chemicals) as a pharmacological agent to inhibit protein synthesis. CHX concentrations of 20 mg/mL were applied to each group of cells, and MYH9 protein expression was measured through western blot analysis at 0, 4, 8, and 12 hours after CHX treatment.

### Ubiquitination assay

Transfection involved the use of HA-tagged ubiquitin vectors (HA-Ub) and His-tagged MYH9 vectors (MYH9-His), with and without the presence of the SP3-461aa overex-pression vector. Following a 24-hour incubation period, the cells were subjected to a 10 mM MG132 treatment (Selleck Chemicals) for a duration of 6 hours. Cell lysates were obtained through the utilization of lysis buffer, followed by the implementation of immunoprecipitation, The lysates were incubated with a His antibody and Protein A/G beads (Santa Cruz Biotechnology) at 4 °C for an extended period of time. Protein analysis was conducted using the western blotting technique.

### Extraction of RNA and RT-qPCR

The TRIzol Reagent (Invitrogen, USA) was utilized for the purpose of extracting total RNA from tissues or cells. Following this, the synthesis of cDNA was conducted utilizing the Prime Script RT Reagent Kit. (Takara, Shiga, Japan). The ChamQ Universal SYBR qPCR Master Mix (Vazyme #Q711) was employed for RT-qPCR, with Actin serving as the reference gene. The specific primer sequences can be found in Table S2.

### Isolation of proteins and western blot analysis

A RIPA (Radio-Immunoprecipitation Assay) buffer supplemented with PMSF was used for cell lysis. Subsequently, Proteins were separated on 10% SDS-PAGE gels and transferred to PVDF membranes. Incubation with specific primary antibodies was performed overnight at 4 °C after the membrane was blocked with 5% nonfat milk so-lution. Subsequently, the blots were washed and exposed to secondary antibodies linked with horseradish peroxidase for a duration of 2 hours. Pro-lighting horseradish peroxidase (HRP) reagent was used to detect protein bands. β-actin and GAPDH were utilized as loading references. The antibodies utilized in this investigation are recorded in Table S3, while the depiction of western blot band densities quantification can be found in Figure S2.

### Animal experiments

BALB/c nude mice, aged between 4 and 6 weeks, were obtained from GemPharmatech LLC. The Animal Care Committees of Wuhan University Medical Research Institute (the Wuhan University Center for Animal Experiment/ABSL-III Laboratory) granted approval for all mouse-related procedures (approval number MRI2022-LAC103). Furthermore, the Institutional Animal Care and Use Committee of Wuhan University approved all animal experiments, which were conducted in accordance with established guidelines. Within the framework of the investigation on tumor growth, a total of six mice were included in each group. The mice were subjected to subcutaneous administration of either OS-RC-2 cells with stable circSP3 knockdown or control cells, with a quantity of 5×106 cells per mouse, specifically targeting the left axillary region. Tumor dimensions were assessed on a weekly basis, and upon completion of a four-week period, the mice were humanely euthanized, with subsequent documentation of tumor masses. In order to establish an *in vivo* metastasis model, a total of 2×10^6^ OS-RC-2 cells with stable circSP3 knockdown or control cells were intravenously injected into 4-week-old BALB/c nude mice via the tail vein. At the conclusion of the six-week period subsequent to injection, A phosphate-buffered formalin solution was used to preserve the lungs of the mice after they were euthanized. In addition, tissue sections were created for each lung block, and nodules associated with metastatic disease were counted.

### Immunohistochemical staining and hematoxylin-eosin staining

Tissue samples, with a thickness of 5 μm and embedded in paraffin, were subjected to staining with hematoxylin and eosin, followed by subsequent analysis using immunohistochemistry. The primary antibody for Ki-67 (Proteintech) was employed at the proper dilution in the experiments. Images were recorded using a Leica DMI4000 B microscope (Leica Microsystems GmbH, Germany) along with NIS-Elements software.

### Statistical analysis

The statistical analysis encompassed the utilization of the Chi-square test and Fisher's exact test to assess significant disparities between two experimental cohorts, while comparisons among multiple groups were conducted through the employment of one-way analysis of variance (ANOVA). The data demonstrated a normal distribution in the statistical analyses, with uniform variances. A p-value less than 0.05, in a two-tailed test, was deemed to be statistically significant. Mean values ± standard deviation (SD) was obtained from a minimum of three independent experiments.

## Results

### Identification and description of circSP3 in ccRCC cells and tissue samples

**Figure 1:**
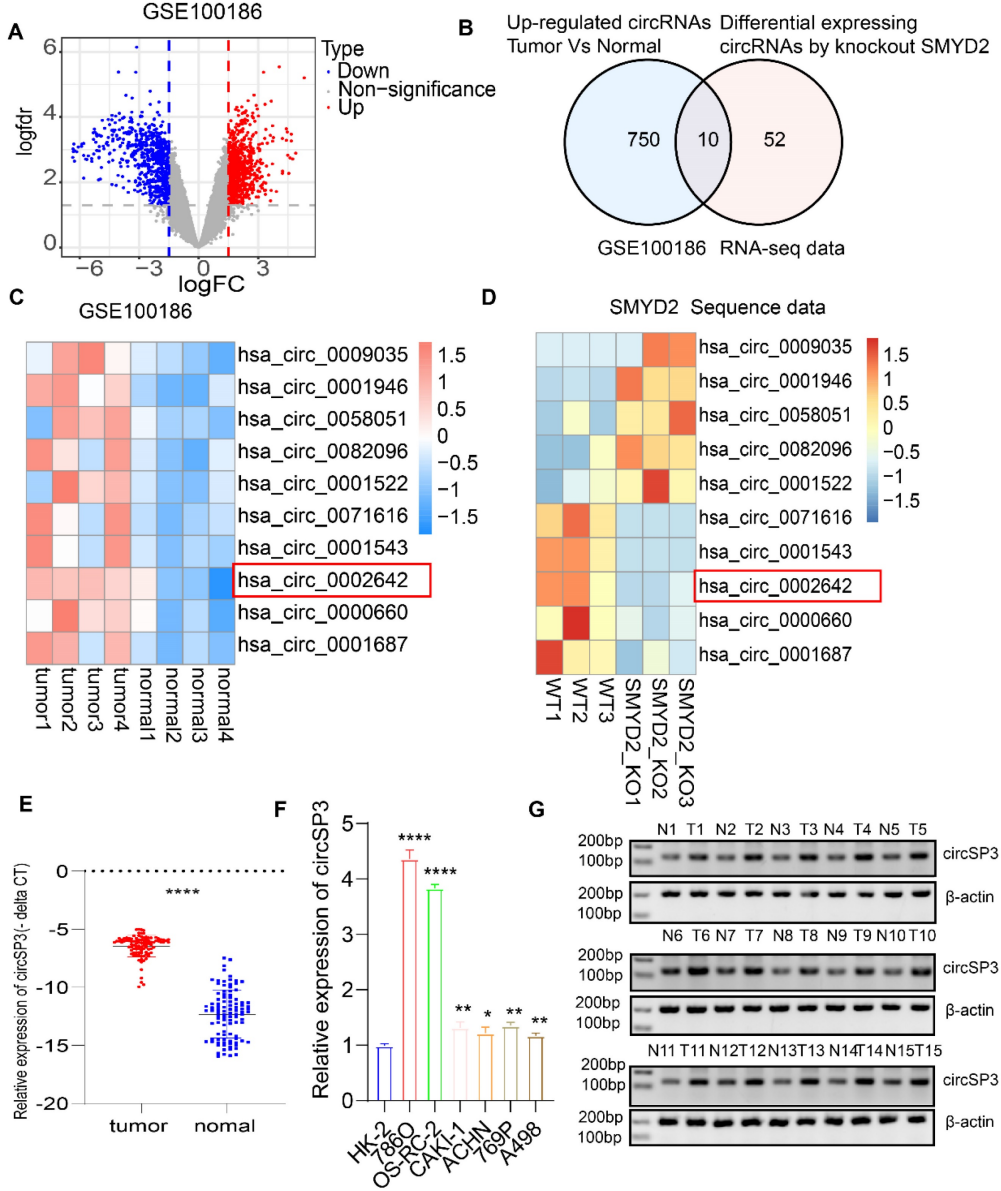
** Identification of circSP3 as a potential oncogenic driver in ccRCC. A.** Volcano Plot of GSE100186 illustrating differentially expressed circRNAs between primary tumors and adjacent normal tissues. **B.** Venn diagram illustrating the comparison between upregulated circRNAs in GSE100186 and differentially expressed circRNAs in SMYD2-deficient RNA-seq data. **C.** Heatmap illustrating the 10 dysregulated circRNAs in ccRCC in comparison to adjacent noncancerous tissues. **D.** Heatmap illustrating the 10 dysregulated circRNAs between wild-type (WT) and SMYD2 knockout (KO) 786O cells. **E.** CircSP3 was upregulated in ccRCC compared to adjacent noncancer tissues (n = 100). **F.** The expression of circSP3 was examined in ccRCC cells and human renal proximal tubular epithelial HK2 cells. **G.** CircSP3 levels were higher in 15 ccRCC compared to paired adjacent noncancerous tissues. Bars represent mean ± SD. *P<0.05, **P<0.01, ***P<0.001.

For a better understanding of how SMYD2 affects ccRCC, we make use of crispr-cas9 gene editing technology to knock it out in ccRCC 786O cells. Then we ran it through full transcriptome sequencing. A total of 72 differential expression circRNAs were identified. GEO microarray datasets containing four matched ccRCC tumor and normal adjacent epithelial tissue samples were analyzed simultaneously for expression profiles of circRNAs (GSE100186; https://www.ncbi.nlm.nih.gov/geo), GSE100186 identified 750 circRNAs that were upregulated. Ten circRNAs were obtained by intersecting the two data sets (Figure 1A-D). Following this, we analyzed the expression of these 10 circRNAs in a range of cancer types, identifying has_circ_0002642 (circSP3) as likely to be an oncogene (Figure S1A). A total of 100 matched pairs of tumor and normal adjacent epithelial tissue were analyzed to explore the role of circSP3 in ccRCC, it was discovered that tumor tissues express circSP3 at a higher level than normal adjacent epithelial tissues (Figure 1E). The levels of circSP3 in the RCC cell lines, 786O, OS-RC-2, CAKI-1, ACHN, A498, and 769P were significantly higher than those of human renal proximal tubular epithelia cell line HK2 (Figure 1F). There was increased expression of circSP3 (hsa_circ_0002642) in ccRCC cells, suggesting that circSP3 might be involved in ccRCC progression. Gel electrophoresis (Figure 1G) demonstrated the consistent overexpression of circSP3 in tumor samples compared to normal tissues, with β-actin serving as a loading control. These findings suggest that circSP3 is significantly up-regulated in ccRCC and may play a crucial role in its progression. In this study, 100 patients diagnosed with clear cell renal cell carcinoma (ccRCC) were stratified into two cohorts based on their circSP3 expression levels, with the aim of investigating the potential involvement of circSP3 in the pathogenesis of the disease. Table 1 illustrates the association between the clinicopathological features and circSP3 expression in a cohort of 100 ccRCC patients. Notably, a significant correlation was observed between the levels of circSP3 expression and the histological grade.

**Figure 2:**
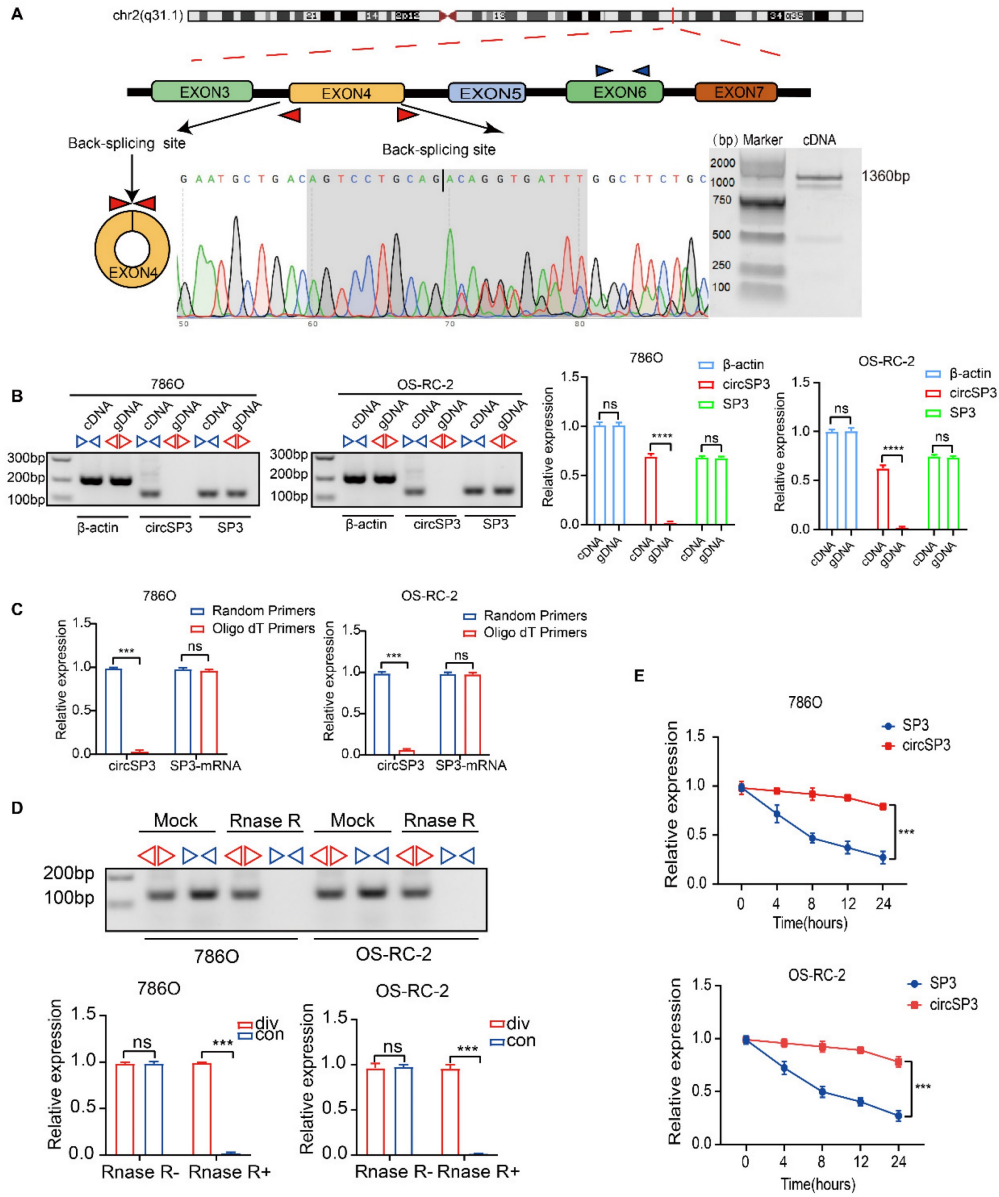
** Characterization of circSP3 in ccRCC. A.** Schematic diagram depicting the genomic loci of circSP3, as well as the divergent and convergent primers specific for circSP3. After performing PCR amplification using the specified divergent flanking primers, Sanger sequencing was conducted to validate the "head-to-tail" splicing of circSP3 in 786O cells.** B.** CircSP3 was identified in complementary DNA (cDNA) through RT-PCR employing divergent or convergent primers, while its presence was not detected in genomic DNA (gDNA) (n = 3). Relative expression levels of circSP3 and SP3 are shown on the right. (ns: not significant, ****p < 0.0001) **C.** CircSP3 and SP3 mRNA levels in ccRCC cells were analyzed and normalized with random primers and oligo dT primers (n = 3). **D.** The effects of RNase R on circSP3 were examined with RT-PCR utilizing divergent or convergent primers (n = 3). **E.** The effects of actinomycin D on circSP3 and SP3 expression were examined with RT-PCR (n = 3). Bars represent mean ± SD. *P<0.05, **P<0.01, ***P<0.001.

CircSP3 (hsa_circ_0002642), derived from exon 4 of the SP3 gene situated at chromosomal location chr2:174819600-174820960 (q31.1), undergoes a back-splicing process, resulting in the formation of a mature sequence that consists of 1360 nucleotides. (Figure 2A). Specific convergent primers (blue) and divergent primers (red) were designed for circSP3. A RT-qPCR product of circSP3 was sequenced for head-to-tail splicing verification using Sanger sequencing. CircSP3 is derived from exons 4 of SP3 according to the circBase database (http://circrna.org/) (Figure 2A, lower panel). In order to verify the presence of an endogenous circRNA, we designed divergent and convergent primers that focused exclusively on the backspliced and canonical forms of SP3 exons 4. RT-PCR with divergent primers detected circSP3, but convergent primers amplified SP3 mRNA (Figure 2B). In addition, divergent primers did not produce any genomic DNA amplified, eliminating the possibility of genomic rearrangement artifacts. Due to the lack of a polyadenylated 3′ tail on circRNA, a random primer or an oligo dT primer was used to detect the presence of circSP3 in reverse transcription products (Figure 2C). After treatment with RNase R, the circSP3 was found to be resistant to this enzyme (Figure 2D). Actinomycin D assays consistently showed that circSP3 transcripts had a longer half-life than linear SP3 transcripts in ccRCC cells, indicating that they were more stable (Figure 2E).

### Knockdown of circSP3 inhibited proliferation, migration, and invasion of ccRCC cells

**Figure 3:**
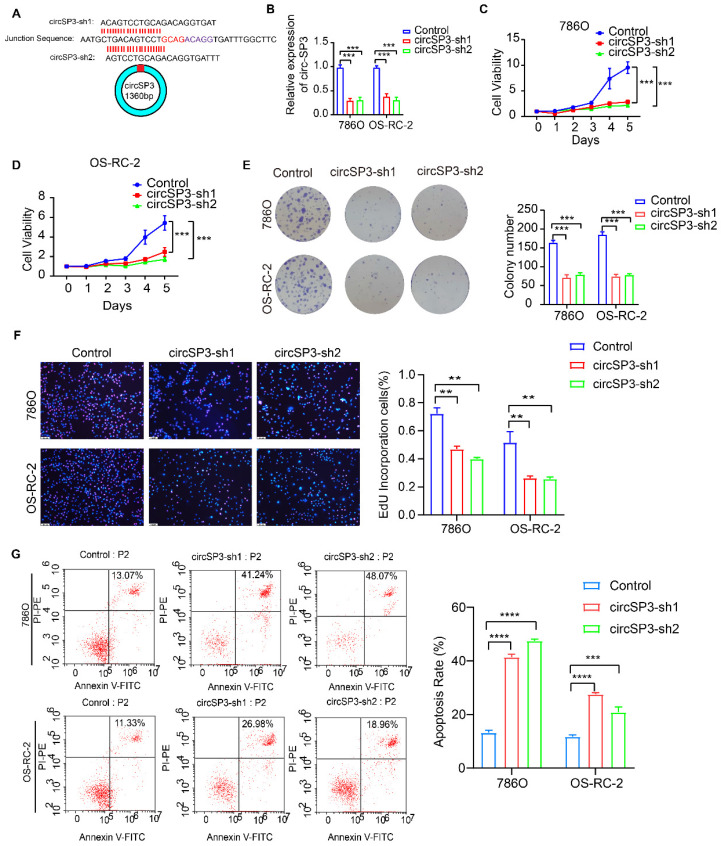
** Effect of CircSP3 Knockdown on ccRCC Cell Viability, Proliferation, and Apoptosis. A.** Design of circSP3 shRNA constructs (circSP3-sh1, circSP3-sh2) and circSP3 junction sequence. **B.** circSP3 expression in 786O and OS-RC-2 cells with control, circSP3-sh1, and circSP3-sh2. (***p < 0.001) **C.** Cell viability in 786O and OS-RC-2 cells over 5 days with control, circSP3-sh1, and circSP3-sh2. (***p < 0.001) **D.** Cell viability in OS-RC-2 cells over 5 days with control, circSP3-sh1, and circSP3-sh2. (***p < 0.001) **E.** Colony formation in 786O and OS-RC-2 cells with control, circSP3-sh1, and circSP3-sh2. Colony numbers on the right. (***p < 0.001) **F.** EdU assays showing proliferative cells in 786O and OS-RC-2 cells with control, circSP3-sh1, and circSP3-sh2. EdU positive cells (%) on the right. (**p < 0.01) **G.** Apoptosis in 786O and OS-RC-2 cells with control, circSP3-sh1, and circSP3-sh2. Apoptosis rate (%) on the right. (****p < 0.0001) Bars represent mean ± SD. *P<0.05, **P<0.01, ***P<0.001.

**Figure 4:**
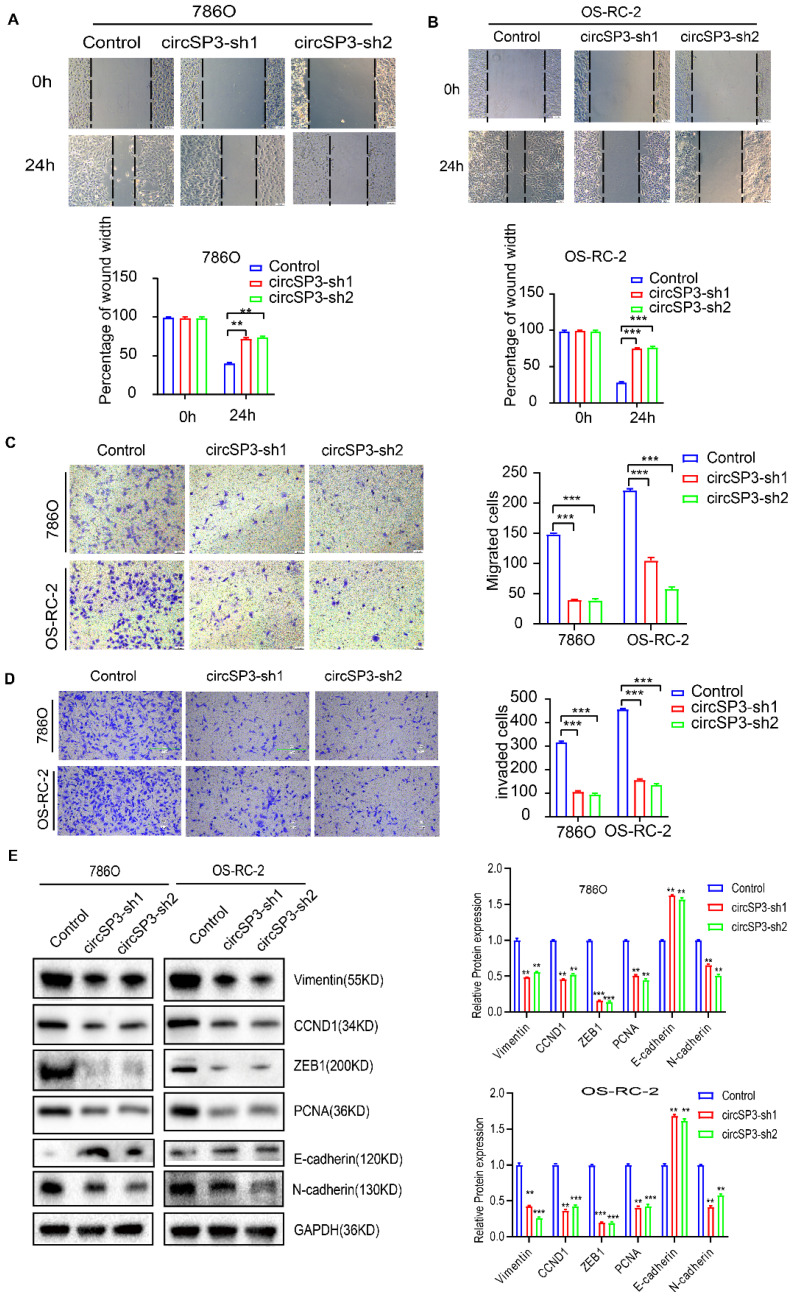
** Effects of circSP3 Knockdown on Migration, Invasion, and Protein Expression in ccRCC Cells. A.** Wound healing assays showing migration in 786O cells with control, circSP3-sh1, and circSP3-sh2 at 0h and 24h. Quantification of wound width is shown below. (**p < 0.01)** B.** Wound healing assays showing migration in OS-RC-2 cells with control, circSP3-sh1, and circSP3-sh2 at 0h and 24h. Quantification of wound width is shown below. (***p < 0.001) **C.** Transwell migration assays in 786O and OS-RC-2 cells with control, circSP3-sh1, and circSP3-sh2. Quantification of migrated cells is shown on the right. (***p < 0.001) **D.** Transwell invasion assays in 786O and OS-RC-2 cells with control, circSP3-sh1, and circSP3-sh2. Quantification of invaded cells is shown on the right. (***p < 0.001) **E.** Western blot analysis of protein expression (Vimentin, CCND1, ZEB1, PCNA, E-cadherin, N-cadherin, GAPDH) in 786O and OS-RC-2 cells with control, circSP3-sh1, and circSP3-sh2. Relative protein expression levels are shown on the right. (**p < 0.01, ***p < 0.001) Bars represent mean ± SD. *P<0.05, **P<0.01, ***P<0.001.

To investigate circSP3's role in clear cell renal cell carcinoma (ccRCC), we reduced its expression using two distinct shRNA constructs. The specifics of these shRNA sequences, their targeted sites, and their efficacy in reducing expression are presented in Figure 3A-B. We utilized a suite of assays, including the Cell Counting Kit-8 (CCK8), colony formation in flat plates, and the EdU incorporation test, to demonstrate that diminishing circSP3 levels markedly hampers cell proliferation, as shown in Figure 3C-F. The results from the Annexin V-FITC Apoptosis Detection assay indicated that the silencing of circSP3 enhances cellular apoptosis, as depicted in Figure 3G. Wound healing assays and transwell assays revealed a significant decrease in migration and invasion of ccRCC cells following knockdown of circSP3 (Figure 4A-D). Subsequent to the analysis of migration, invasion, and cell cycle-associated proteins, it was observed that the knockdown of circSP3 led to a reduction in cell migration and invasion, and induced cell cycle arrest (Figure 4E). In summary, the silencing of circSP3 was observed to inhibit cell proliferation, migration, and invasion in ccRCC cells under *in vitro* conditions.

**Figure 5:**
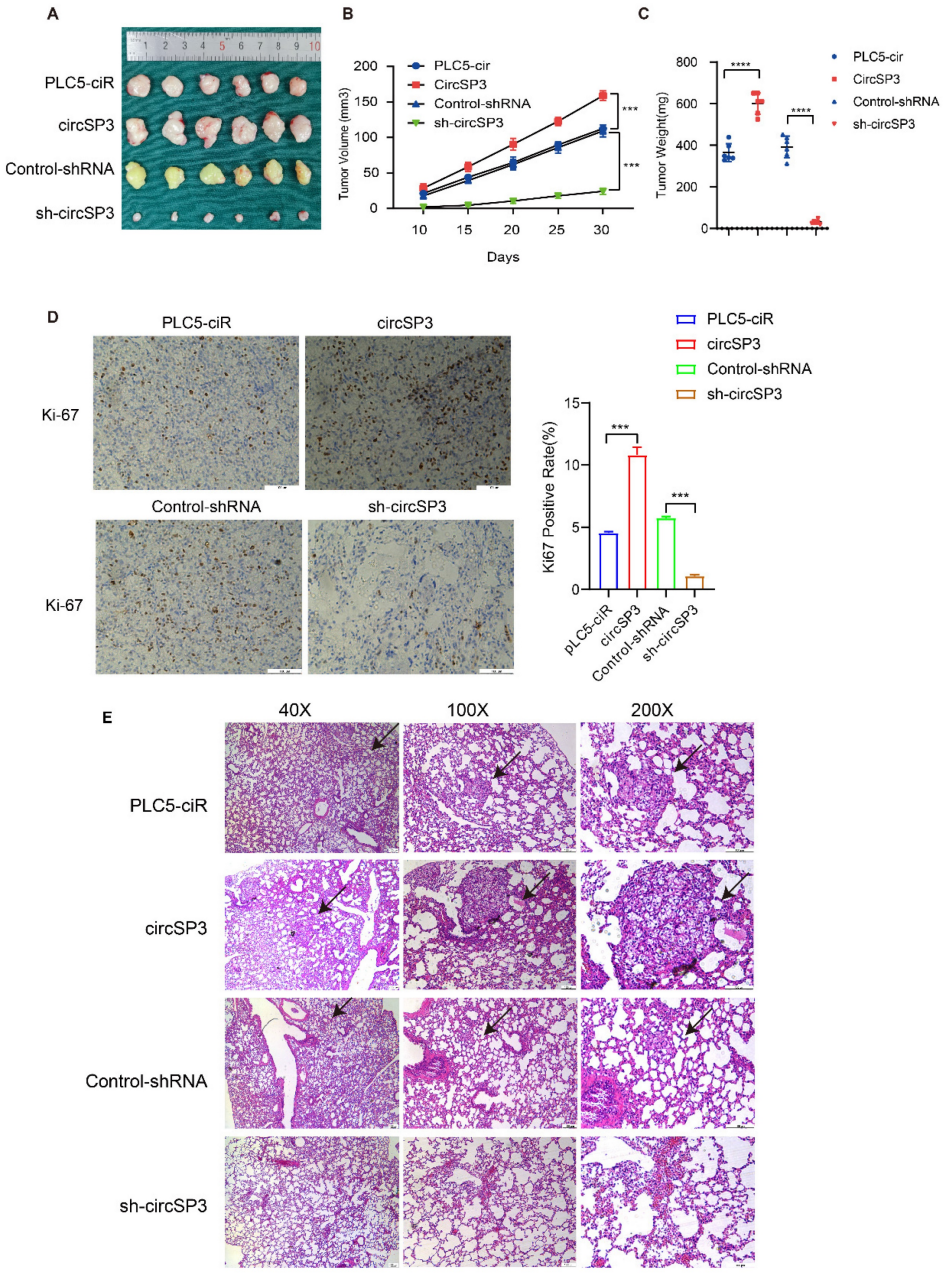
** CircSP3 promotes the proliferation, invasion, and metastasis of renal clear cell carcinoma cells *in vivo*. A-C.** Schematic illustration depicting the terminal phase of xenograft tumors in BALB/c nude mice. The alterations in tumor volume were documented at 5-day intervals subsequent to the initial 10-day duration. The weights of xenograft tumors were measured and recorded at the conclusion of the research study. **D.** Xenograft tumors were immunohistochemically stained for Ki67 expression. **E.** The lungs extracted from mice injected with circSP3, circSP3-sh1 or control vector transfected cells were subjected to H&E staining analysis (n = 5). Bars represent mean ± SD. *P<0.05, **P<0.01, ***P<0.001.

To investigate the *in vivo* tumorigenic and metastatic function of circSP3, OS-RC-2 cells were transfected with circSP3-OV and sh-circSP3 vectors, along with their respective Control vectors. Following subcutaneous seeding of the cell lines, tumor volume was measured 10, 15, 20, 25, and 30 days later. PLC5-ciR tumor sizes were significantly smaller than those seen in the circSP3-OV group. Similarly, the sh-circSP3 group displayed significantly smaller tumors compared to the Control-shRNA group (Figure 5A-C). Immunohistochemical (IHC) analysis was conducted to assess the expression of the proliferation marker Ki67 in the xenograft tumors. Notably, the overexpression of circSP3 resulted in a significant upregulation of Ki67 staining (Figure 5D). Furthermore, the application of Hematoxylin and eosin (H&E) staining provided confirmation of a noteworthy escalation in metastasis within the circSP3-OV group when compared to the plc5-ciR group (Figure 5E).

### CircSP3 encodes a novel 461-amino acid peptide referred to as SP3-461aa

**Figure 6:**
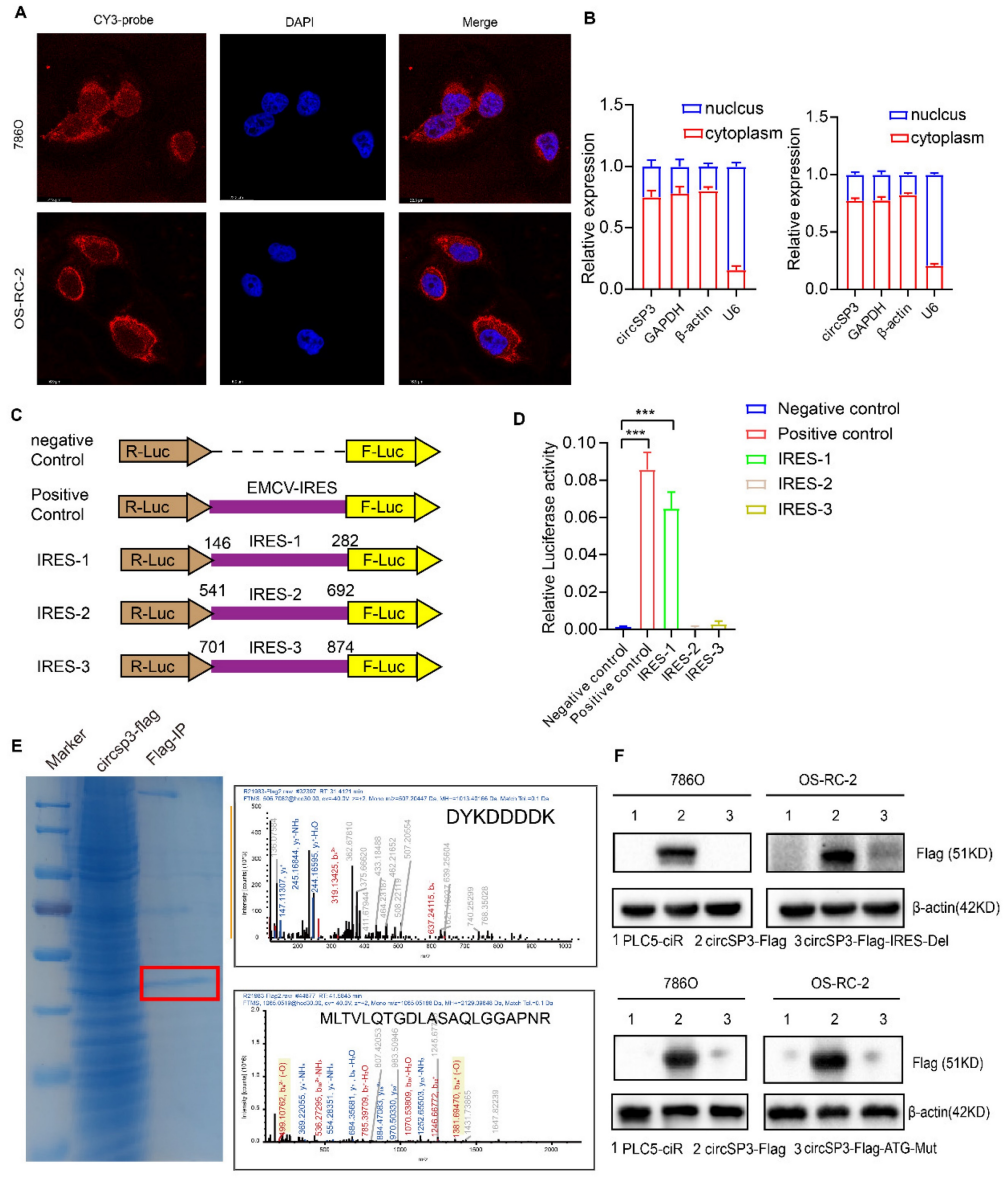
** CircSP3 coded a novel 461-amino acid peptide named as SP3-461aa. A.** The subcellular localization of circSP3(red, CY3-probe) and nuclei (blue, DAPI) in ccRCC cells was assessed by FISH using junction-specific probes. **B.** The distribution of circSP3 within the nuclear and cytoplasmic compartments of ccRCC cells (n = 3). **C-D.** IRES sequences and truncation mutants were incorporated into the Luc2-IRES-Report vector. The purported IRES functionality of circSP3 was assessed in 293T cells (n =3) **E.** Total proteins were extracted from 293T cells transfected with the circSP3-FLAG vectors, followed by immunoprecipitation using the FLAG-tag antibodies. The amino acid sequences of SP3-461aa were determined by LC-MS. **F.** The SP3-461aa-FLAG proteins were detected using FLAG antibodies. IRES deletion and ATG mutation attenuated SP3-461aa expression. Bars represent mean ± SD. *P<0.05, **P<0.01, ***P<0.001.

CircSP3 knockdown inhibited proliferation, migration, and invasion of ccRCC cells previously. We explored the possibility that circSP3 encodes a peptide, in light of emerging evidence that some circRNAs are capable of coding proteins 22. We first examined the subcellar location of circSP3 via RNA Fluorescence *in situ* hybridization and Cytoplasmic separation detection. The main location of circSP3 is in the cytoplasm (Figure 6A-B), which indicates its potential molecular mechanism. Upon further analysis of circSP3, it was determined that it contains a 1383nt Open Reading Frame ORF (green), possibly encoding a 461aa peptide. Figure S1C-D illustrates the mature sequences of circSP3 and SP3-461aa. Three putative ribosome entry site sequences were identified through bioinformatics analysis (146-282nt, 541-692nt, and 701-874nt, outlined in purple). 5'-cap-independent coding RNAs require these sequences to initiate translation 23, 24. Subsequently, the three IRES sequences were individually inserted into the Luc2-IRES-Report vector, and their activity was assessed through a dual-luciferase assay (Figure 6C), The Luc2-EMCV-IRES-Report vector was used as a positive control with an EMCV IRES sequence. It was found that the putative 146-282nt IRES sequence would initiate protein translation strongly, whereas the 541-692nt and 701-874nt IRES sequences had little or no effect on protein translation (Figure 6D).

For further assessment of circSP3's protein-coding ability, we constructed a novel vector with FLAG sequences. The endogenous circSP3 junction is situated within the open reading frame (ORF), a FLAG sequence was relocated to the stop codon of the ORF, resulting in circSP3-FLAG (Figure S1E). Subsequently, in 293T cells transfected with circSP3-FLAG, the amino acid sequences of SP3-461aa were determined using liquid chromatography tandem mass spectrometry (LC-MS). Remarkably, A specific protein fragment originating from SP3-461aa (MLTVLQTGDLASAQLGGAPNR) was successfully identified. Thereby providing evidence for the translation of circSP3 into SP3-461aa within cellular milieu (Figure 6E). In addition, a FLAG-labeled vector devoid of the IRES 146-282nt sequence, referred to as circSP3-FLAG-IRES-Del, was synthesized. Subsequently, a Flag tag antibody was used to assess the expression of SP3-461aa-FLAG following introduction of these vectors and their empty corresponding vectors into cells. The results further demonstrate the importance of the IRES sequence in circSP3 translation (Figure 6F, upper panel). Vectors expressing circSP3-ATG-mut were subsequently generated and transfected into ccRCC cells, followed by the assessment of SP3-461aa peptide expression (Figure 6F, lower panel). Subsequently, following the downregulation of circSP3, the expression of the SP3-461aa peptide was assessed using an SP3 antibody. This antibody is characterized by an immunogenic sequence identical to that of the SP3-461aa peptide (Figure S1F). Furthermore, Western blot analysis was conducted on the transfected liner SP3-461aa-Flag vector ccRCC cells, revealing the presence of the specific peptide sequence of SP3-461aa-FLAG in both cell lines (Figure S1G). This finding provides additional evidence supporting the translation of circSP3. Collectively, our findings provide evidence that circSP3 undergoes translation to produce SP3-461aa in a manner reliant on internal ribosome entry site (IRES) activity. Furthermore, we demonstrate the endogenous expression of SP3-461aa in cell lines derived from ccRCC.

### SP3-461aa facilitated the proliferation and metastasis of ccRCC cells

**Figure 7:**
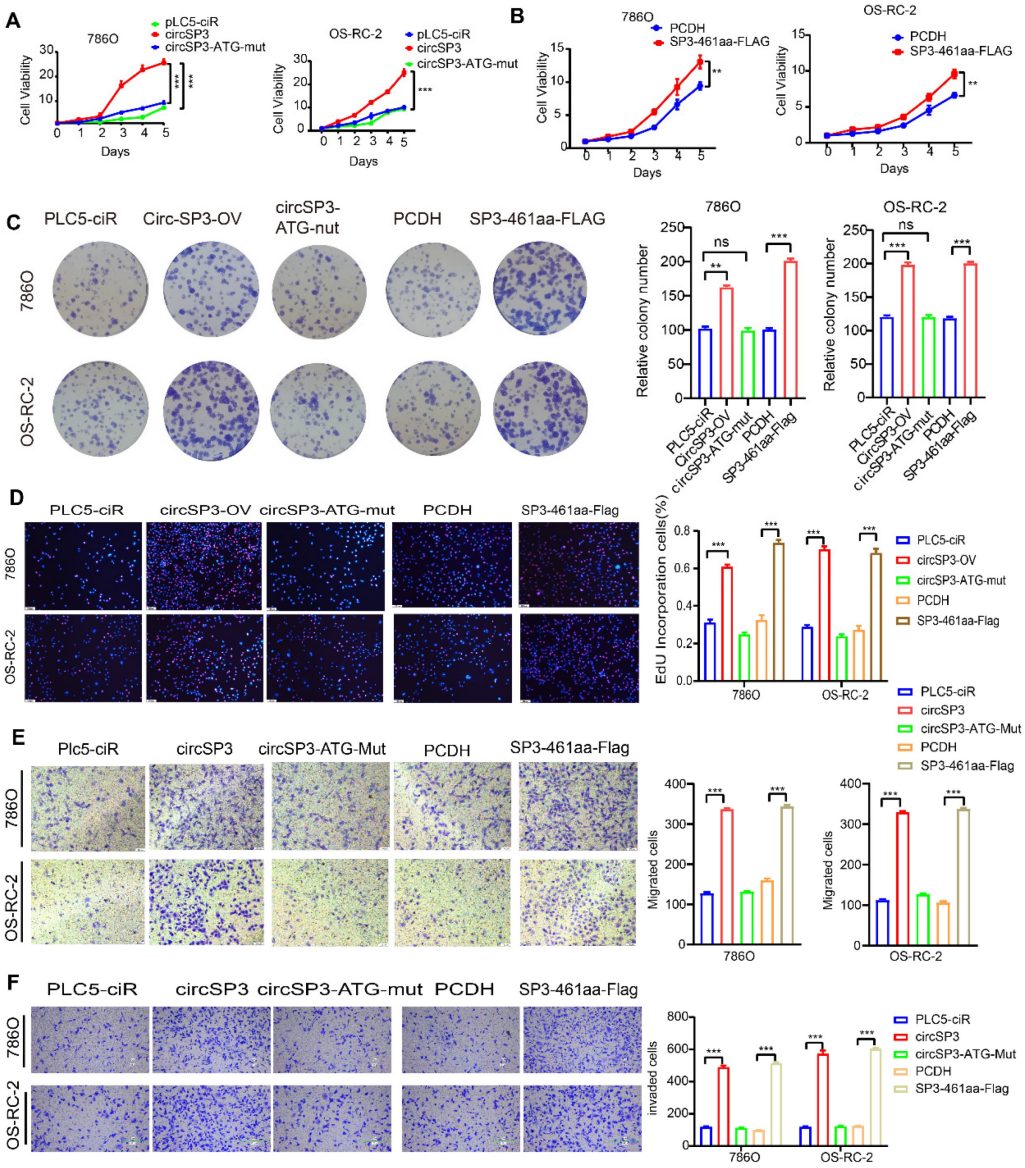
** The SP3-461aa induced ccRCC cell proliferation and metastasis. A-B.** ccRCC cell viability was increased by over-expressing SP3-461aa (n = 5). **C.** SP3-461aa induced colony formation of ccRCC cells (n = 3). **D.** EdU assays demonstrating that SP3-461aa increased ccRCC cell proliferation. **E.** Modified Boyden chamber assays demonstrating that SP3-461aa induced ccRCC cell migration (n = 3). **F.** Modified Boyden chamber assays demonstrating that SP3-461aa induced ccRCC cell invasion (n = 3). Values were expressed as mean ± SD. *P<0.05, **P<0.01, ***P<0.001.

Having established the translation of circSP3 into a novel peptide referred to as SP3-461aa, our investigation delved into the potential contributions of circSP3 or SP3-461aa towards its functions. The ccRCC cells underwent stable transfection with the circSP3-OV, circSP3-ATG-mut, and the empty vector PLC5-ciR, along with the SP3-461aa-FLAG and PCDH-CMV-coGFP vectors. The validation of circSP3 overexpression's efficiency and its impact on SP3 mRNA expression were further confirmed through the execution of RT-qPCR (Figure S1H-I). The proliferation of ccRCC cells was observed to be enhanced by the overexpression of putative circSP3, as depicted in Figure 7A-B. However, this effect was reversed upon mutation of the ATG sequence. Additionally, the proliferation was further augmented with the overexpression of SP3-461aa-FLAG. Both the flat plate colony formation assay and the EdU assay produced similar results (Figure 7C-D). Similarly, Transwell assays showed similar results regarding migration and invasion of ccRCC cells (Figure 7E-F).

### SP3-461aa interacted directly with MYH9, safeguarding it from degradation

**Figure 8:**
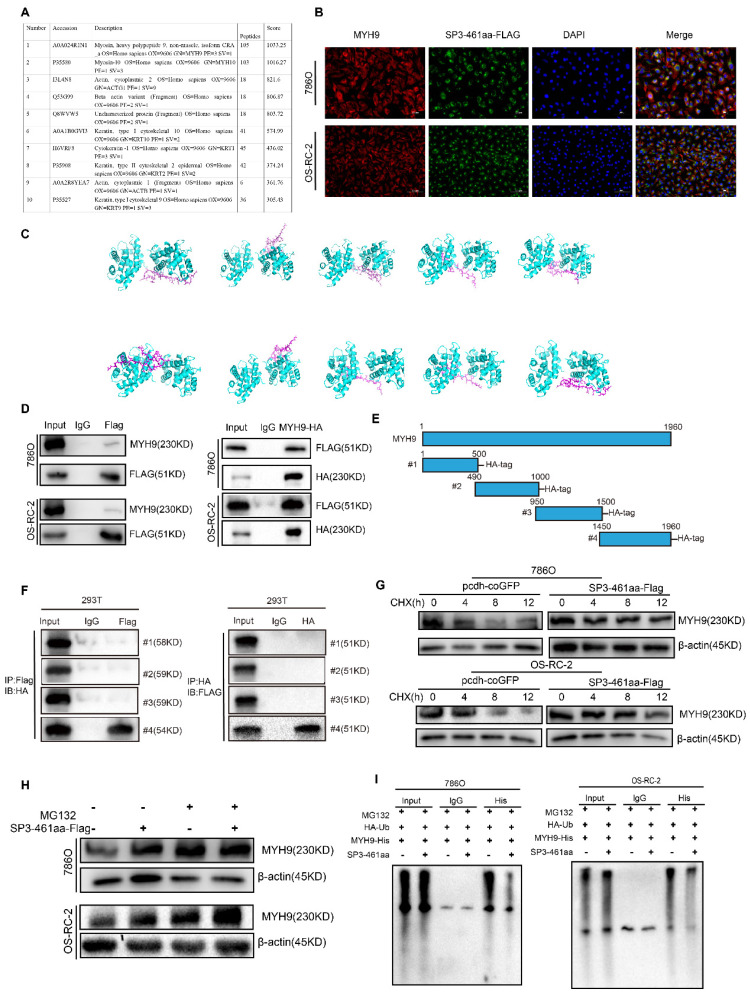
** SP3-461aa directly interacted with MYH9 and prevented its degradation. A.** LC-MS analysis of the immunoprecipitates to identify proteins that may interact with SP3-461aa. **B.** Anti-FLAG and anti-MYH9 antibodies were used to determine the colocalization of SP3-461aa (green) and MYH9 (red) in ccRCC cells. Scale bar, 20 μm. **C.** The conformational modeling of SP3-461aa was conducted using PEP-FOLD, and subsequently, their docking to MYH9 was performed utilizing the ATTRACT2 force field through the PEP-SiteFinder pipeline. The MYH9 complex was visually represented using PyMOL (version 1.8; Schrödinger), incorporating the top 10 peptides. Distinct colors were employed to distinguish each individual peptide. **D.** ccRCC cells were transfected with SP3-461aa-FLAG, followed by immunoprecipitation to evaluate the interaction between SP3-461aa and MYH9. **E.** An illustration showing MYH9 fragmented into four pieces and tagged with HA tags.** F.** Immunoprecipitation was used to test the direct mutual interactions between EIF6-224aa and HA-tagged MYH9 (n = 3). **G.** Treatment of ccRCC cells with 20 mg/mL CHX for varying durations (0, 4, 8, or 12 hours) allowed us to investigate the role of SP3-461aa in maintaining the stability of MYH9 protein. **H.** ccRCC cells were subjected to MG132 treatment at a concentration of 10 mM. SP3-461aa was validated as a factor that influenced MYH9 proteosomal degradation. **I.** HA-Ub, MYH9-His, PCDH or linear-SP3-FLAG were co-transfected into 293T cells. Subsequently, we tested whether SP3-461aa affected the ubiquitination levels of MYH9 protein in the presence of 10 mM MG132.

An investigation into the molecular mechanisms underlying SP3-461aa's tumor-promoting functions, we conducted coimmunoprecipitation experiments in 293T cells overexpressing SP3-461aa-FLAG. The aim was to identify the potential target molecules of SP3-461aa. We used anti-FLAG antibodies for immunoprecipitation on 293T cells transfected with SP3-461aa-FLAG or control cells (Figure 8A). A LC-MS analysis of the precipitates was performed to identify proteins that might interact with SP3-461aa. Among the numerous candidates, MYH9 protein exhibited potential binding with SP3-461aa. Moreover, the immunofluorescence staining analysis revealed a co-localization of SP3-461aa and MYH9 within the cytoplasmic region (Figure 8B), indicating a potential interaction between SP3-461aa and MYH9. The conformational analysis of SP3-461aa was conducted using PEP-FOLD, followed by docking with MYH9 through ZDOCK, in order to examine the possible direct interaction between SP3-461aa and MYH9 25. PyMOL was used as a tool to visualize the top ten proteins complexing with MYH9 (the PyMOL Molecular Graphics System, version 1.8; Schrödinger), with each protein represented in distinct colors. Additionally, the residues at the interface between the proteins and peptides were appropriately labeled. With PyMOL, we visualized the top ten proteins that complex with MYH9 (the PyMOL Molecular Graphics System, version 1.8; Schrödinger). Additionally, the residues at the interface between the proteins and peptides were appropriately labeled (Figure 8C).

Immunoprecipitation was performed to further validate the interaction of SP3-461aa and MYH9. Specifically, 293T cells were co-transfected with MYH9 vectors and SP3-461aa-FLAG vector, followed by the execution of immunoprecipitation assays (Figure 8D). To further examine the interaction between SP3-461aa and MYH9, the MYH9 protein was divided into four fragments with hemagglutinin (HA) tags (Figure 8E). Immunoprecipitation assays were performed with MYH9 truncated vectors as well as SP3-461aa-FLAG vectors cotransfected into 293T cells. As shown in Figure 8F, SP3-461aa interacted primarily with MYH9's fourth fragment. Cycloheximide (CHX), a compound that inhibits protein synthesis, was introduced to ccRCC cells that were transfected with either the SP3-461aa-FLAG vector or PCDH-CMV-coGFP. In order to investigate the effects of SP3-461aa on MYH9 protein levels, MYH9 protein levels were measured at 0, 4, 8, and 12 hours (Figure 8G). The enhanced expression of SP3-461aa resulted in a decrease in the rate of degradation of MYH9, suggesting that the naturally occurring SP3-461aa derived from circSP3 serves as a protective factor against the degradation of MYH9. In addition, Transfected ccRCC cells were treated with MG132 after being transfected with the SP3-461aa-Flag vector and PCDH-CMV-coGFP. After six hours of incubation, the MYH9 protein expression was measured. SP3-461aa overexpression protected the MYH9 protein from proteasomal degradation, as shown in Figure 8H. Following the aforementioned findings, a subsequent investigation was conducted to assess the ubiquitination of the MYH9 protein. Notably, a significant reduction in MYH9 ubiquitination level was observed after overexpression of SP3-461aa, thereby affording protection against proteasomal degradation, as illustrated in Figure 8I.

### SP3-461aa enhanced ccRCC progression through the activation of the MYH9/PI3K/Akt signaling pathway

**Figure 9:**
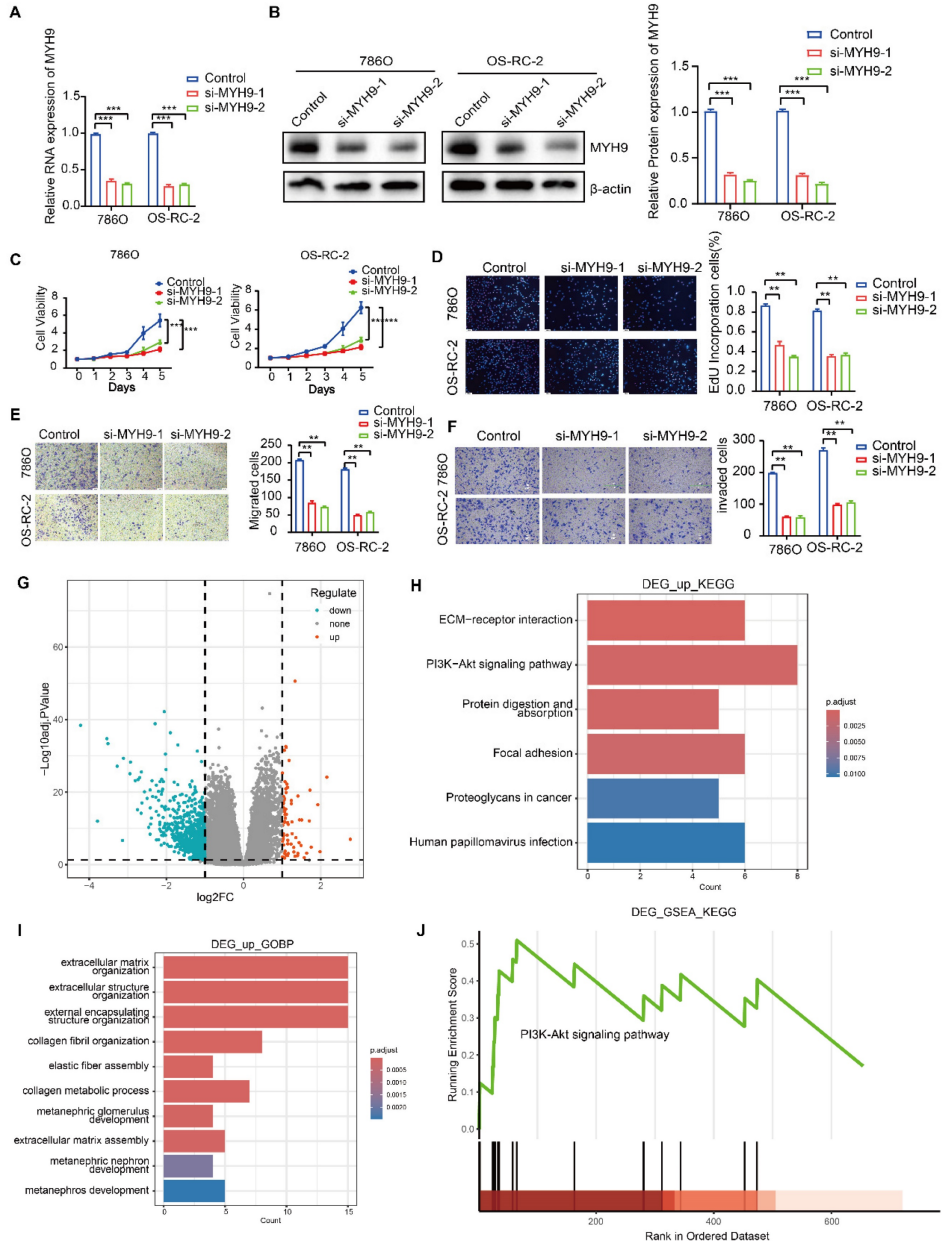
** The function of the MYH9 gene in renal clear cell carcinoma. A-B.** The silencing efficacy of MYH9 expression was evaluated using RT-qPCR and immunoblotting (n = 3).** C-D.** The proliferation of ccRCC cells was evaluated by utilizing EdU assays and CCK-8 assays to investigate the impact of MYH9 knockdown.** E-F.** After conducting MYH9 knockdown, we employed Transwell assays to examine the migratory and invasive capacities of 786O and OS-RC-2 renal cancer cells (n = 3). **G-J.** Volcano plot of differential gene analysis and Gene enrichment analysis result chart. Bars represent mean ± SD. *P<0.05, **P<0.01, ***P<0.001

Two siRNAs were employed to induce knockdown of MYH9 expression, and the efficacy was confirmed in both 786O and OS-RC-2 cell lines through the utilization of RT-qPCR and western blot analysis. These techniques were employed to assess the impact of MYH9 on the oncogenic functions of circSP3/SP3-461aa (Figure 9A-B). The verification of the proliferation, migration, and invasion abilities of MYH9 in ccRCC cell lines was conducted (Figure 9C-F). Subsequently, employing the Kidney Renal Clear Cell Carcinoma (KIRC) dataset from The Cancer Genome Atlas (TCGA) database, we conducted a differential gene expression analysis contingent on the varying expression levels of the MYH9 gene. This analysis resulted in the identification of 736 genes exhibiting upregulation and 72 genes demonstrating downregulation (Figure 9G). We performed an enrichment analysis of differentially expressed genes, and observed that both KEGG (Kyoto Encyclopedia of Genes and Genomes) and GSEA (Gene Set Enrichment Analysis) enrichments were significantly associated with the PI3K-Akt signaling pathway (Figure 9H-J).

**Figure 10:**
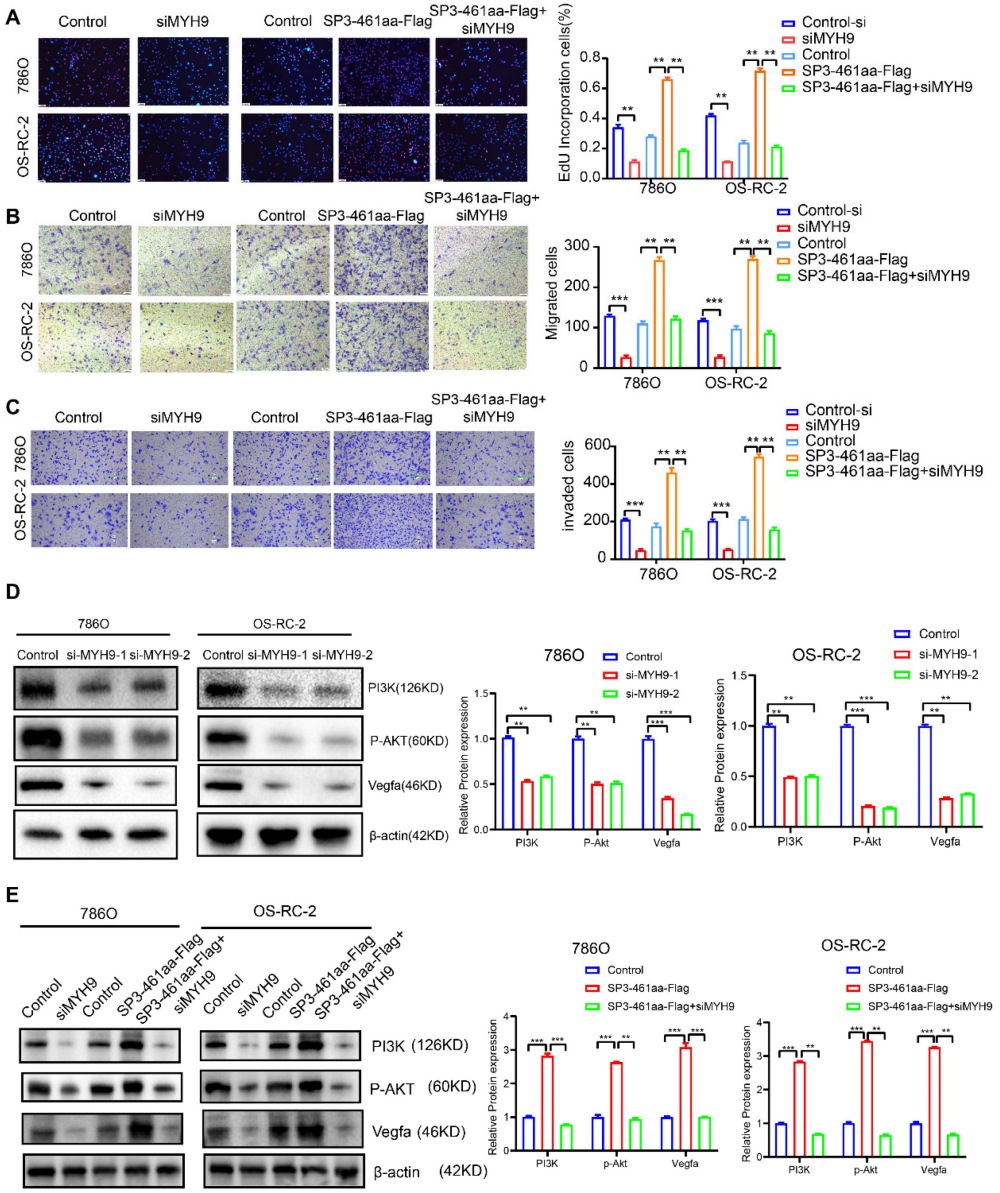
** The stabilization of MYH9 protein by SP3-461aa triggered the activation of the PI3K-Akt signaling pathway. A.** EdU assays were conducted to investigate the proliferation capabilities of ccRCC cells that were transfected with control-si, siMYH9, PCDH-CMV-coGFP, SP3-461aa-FLAG or SP3-461aa-FLAG + siMYH9. **B-C.** The migratory and invasive capacities of ccRCC cells were subsequently examined through modified Boyden chamber assays. **D.** The impact of knockdown of MYH9 on the expression levels of the VEGFA, PI3K- Akt signaling pathways in ccRCC cell lines was assessed.** E.** The impact of control-si, siMYH9, PCDH-CMV-coGFP, SP3-461aa-FLAG or SP3-461aa-FLAG + siMYH9 on VEGFA and PI3K/AKT pathways was assessed in ccRCC cells. Values were expressed as mean ± SD. *P<0.05, **P<0.01, ***P<0.001.

Subsequently, the ccRCC cell lines were transfected with control-si, siMYH9, PCDH-CMV-coGFP, SP3-461aa-FLAG, or SP3-461aa-FLAG + siMYH9, followed by the assessment of their proliferation, migration, and invasion capabilities (Figure 10A-C). The antagonistic effects of MYH9 knockdown on the oncogenic properties of SP3-461aa were observed, leading to a significant inhibition of cellular proliferation, migration, and invasion capabilities. These findings suggest that MYH9 serves as a functional target of SP3-461aa. Additionally, prior research has demonstrated a correlation between MYH9 expression and the activation of the VEGFA pathway 26. The effects of SP3-461aa overexpression and MYH9 knockdown on the PI3K/Akt pathway were examined. The overexpression of SP3-461aa resulted in an upregulation of the expression of PI3K/Akt proteins, whereas the knockdown of MYH9 using siRNA led to a downregulation of their expression (Figure 10D-E).

Our study has elucidated that circSP3 (hsa_circ_0002642) engages in an interaction with ribosomal components, culminating in the translation of a novel peptide designated SP3-461aa. This peptide interacts directly with MYH9, leading to a decreased ubiquitination of MYH9 and consequently inhibiting its proteasomal degradation. This interaction subsequently facilitates the activation of the PI3K-Akt signaling pathway, which enhances the oncogenic potential of circSP3. We further examined the expression of MYH9 in clear cell renal cell carcinoma (ccRCC) tissues and adjacent normal renal tissues, along with the activation of PI3K and AKT. The results indicated an increased expression of MYH9 in ccRCC tissues, which was associated with the activation of the PI3K-AKT pathway (Figure S2A). In xenografted samples, we assessed the expression of MYH9, PIK3, and AKT. The results demonstrated that the overexpression of circSP3 significantly increased the expression of MYH9 and activated the expression of PI3K and AKT (Figure S2B).

## Discussion

Over the past several decades, extensive research has shed light on the pivotal role of SMYD2 in various cancer types, including lung adenocarcinoma, cervical cancer, triple-negative breast cancer, and pancreatic ductal adenocarcinoma. This growing body of evidence highlights SMYD2 as a key molecular player in the oncogenic processes across these diverse malignancies 4, 5, 27-29. In our previous study, we identified an upregulation of SMYD2 in renal cell carcinoma, which was significantly correlated with adverse clinical prognoses 10. However, further investigation is warranted to provide a comprehensive understanding of the involvement of SMYD2 in renal cell carcinoma.

Histone-modifying enzymes are crucial regulators of chromatin-associated processes, particularly transcription. In addition to their role in histone modification, these enzymes exert control over gene expression by modifying nonhistone proteins, including transcription factors 4, 24, 25. Depletion of the DHX9 protein increases the production of circular RNA 30. In the present investigation, it was observed that SMYD2 possesses the ability to modulate the expression of circRNAs. To identify potential functional circRNAs, we conducted an initial screening of a set of circRNAs that displayed dysregulation in four paired tumor/normal adjacent epithelial tissue samples, along with SMYD2-knockout (SMYD2-KO) cell lines. Among these, we have identified 10 circular RNAs (circRNAs) that exhibit statistically significant differential expression. As a result, circSP3 was selected for comprehensive examination based on its association with the clinicopathological characteristics of ccRCC patients. circSP3 originates from chromosome 2q31.1 and has been linked to the initiation of hepatocellular carcinoma 31. A subsequent inquiry into the protein-coding capacity of circSP3 has recently unveiled that the internal ribosome entry site (IRES) of synthetic circRNAs allows them to undergo translation cap-independently. In order to ascertain the existence of an Internal Ribosome Entry Site (IRES) structure within the circSP3 sequence, we performed analyses utilizing circRNADB and IRESfinder. This investigation led to the identification of three potential IRES sequences, thereby substantiating its ability to encode proteins. Subsequent experimental findings have substantiated the occurrence of IRES-mediated translation initiation, thereby unveiling the presence of a hitherto unknown 461-amino acid peptide encoded by circSP3. Numerous studies have documented the atypical expression patterns of non-coding RNAs (ncRNAs) across cancer types in recent decades. It encompasses microRNAs (miRNAs), long non-coding RNAs (lncRNAs), and circular RNAs (circRNAs). Researchers have recently discovered that miRNAs and long noncoding RNAs play pivotal roles in the formation of tumors and the development of metastases. CircRNAs, which represent a prominent subclass of RNA within the human transcriptome, have garnered significant attention as a subject of investigation. In addition to their role in regulating gene expression or development through miRNA adsorption, circRNAs have demonstrated significant importance in human malignancies. The predominant function of circRNAs is widely acknowledged to be their capacity as miRNA sponges; however, it is imperative to acknowledge that only a restricted subset of circRNAs possess flawless multiple miRNA binding sites, in turn, this raises the possibility that circRNAs have functions outside of being miRNA sponges.

Recent research has provided evidence for the existence of functional peptides that are produced from small open reading frames (sORFs) located within non-coding RNAs (ncRNAs), including primary transcripts containing hairpin structures (pri-miRNAs), long non-coding RNAs (lncRNAs), and circular RNAs (circRNAs). It suggests that these non-coding RNAs (ncRNAs) have been undervalued for their coding capacity. As a result of their distinctive circular structure, protein-coding circular RNAs (circRNAs) may show enhanced stability in protein expression. In various types of cancer, circular RNAs (circRNAs) and their ensuing protein products have been shown to exert significant effects, such as liver cancer, glioblastoma, and colon cancer 18, 32, 33. An illustrative example is the role of circFBXW7 in inhibiting the development of glioma tumors through the production of FBXW7-185 aa, this reduces c-Myc stabilization by opposing the USP28-mediated stabilization process. Nevertheless, there is a limited understanding of the significant contribution of protein-coding circRNAs to clear cell renal cell carcinoma (ccRCC). *In vitro* and *in vivo* assays were performed to investigate the effect of circSP3 on malignant behavior in ccRCC cells. After the downregulation of circSP3 expression in both 786O and OS-RC-2 cell lines, a significant decrease in cell proliferation, migration, and invasion capabilities was observed (Figure 3C-G and Figure 4A-D). After confirming the encoding of SP3-461aa by circSP3, additional evaluations were conducted to determine the specific contribution of either circSP3 or SP3-461aa to the oncogenic functions. In order to investigate the functions of SP3-461aa, we constructed a circSP3-OV without the IRES and conducted a comparative analysis with a hypothetical circSP3-OV, considering the significant role of IRES in circRNA translation. According to the illustration presented in Figure 5, it was observed that SP3-461aa, as opposed to circSP3, exhibited a significant influence on the proliferation and metastasis of ccRCC cells.

Through additional investigation into the mechanism of SP3-461aa, it has been substantiated that SP3-461aa exhibits interaction with the MYH9 protein. Myosin II subfamily member MYH9 plays an important role in cell adhesion, cytokinesis, and morphogenesis 34-36. MYH9 encodes non-muscle myosin IIA (NMIIA), which has been extensively demonstrated to contribute to cancer progression, including cell proliferation, invasion, and metastasis. Based on prior research findings, in skin, head and neck squamous cell cancers as well as tongue squamous cell cancers, MYH9 has been identified as a tumor suppressor 37-39. In contrast, recent reports indicate that MYH9 functions as an oncogene in gastric, colorectal non-small cell lung, nasopharyngeal carcinoma, and esophageal squamous cell cancers 40-44. In the context of clear cell renal cell carcinoma (ccRCC), Several studies have examined MYH9's role in tumor metastasis. Such as, Xu et.al. documented that the pharmacological inhibition of NMMHC-IIA using blebbistatin effectively suppressed the nuclear translocation of CXCR4, thereby attenuating the metastatic potential of renal cell carcinoma (RCC) cells 45. A separate study demonstrated that MYH9 facilitates the progression of renal cell carcinoma and resistance to sunitinib through AKT signaling 46. In this study, using ubiquitin-mediated degradation pathways, we found that the direct interaction between SP3-461aa and MYH9 effectively prevented MYH9 protein degradation. According to these findings, MYH9 is an ideal target for SP3-461aa. Recent research has demonstrated that the interaction between MYH9 and GSK3b leads to a decrease in the expression of the latter protein via ubiquitin-mediated degradation. Following this event, the Wnt/beta-catenin pathway is activated, resulting in downstream oncogenic tumor phenotypes 47. Recent research has indicated that biomarkers for renal carcinoma can be detected in urine samples 48. Notably, a study conducted by Vincenzo Petrozza and colleagues has identified secreted miR-210-3p as a promising non-invasive biomarker for clear cell renal cell carcinoma 49. Incorporating the Multi-Omic Subtype (MoS) classification by Meng *et al.* into our research to understand how circSP3 and SP3-461aa expression varies across different ccRCC subtypes. This could provide insights into personalized therapeutic approaches based on MoS classification 50. In future research, we can also attempt to detect circSP3 in urine samples to further evaluate its relationship with the prognosis of renal cancer. In the current investigation, the interplay of SP3-461aa and MYH9 was found to trigger the activation of the PI3K-Akt signaling pathway, thereby facilitating the proliferation and metastasis of ccRCC cells.

The main limitations of our study are the need for further elucidation of the detailed molecular mechanisms involved in the interaction between SP3-461aa and MYH9 and their downstream effects on the PI3K-Akt signaling pathway. This includes exploring other potential interacting partners and additional signaling pathways influenced by circSP3 and SP3-461aa. Additionally, while our findings suggest that circSP3 could serve as a prognostic biomarker, further validation in larger clinical cohorts is essential. Investigating the stability and detectability of circSP3 in biofluids (e.g., blood, urine) will be crucial to evaluate its utility as a non-invasive biomarker.

In conclusion, our research provides significant evidence supporting the critical function of the peptide SP3-461aa, produced by the circular RNA circSP3, in enhancing both the proliferative and metastatic potentials of clear cell renal cell carcinoma (ccRCC) cells. Importantly, this study delineates the involvement of SP3-461aa in activating the MYH9/PI3K-Akt signaling pathway, offering insights into the molecular dynamics of ccRCC progression. Furthermore, our results suggest the potential of circSP3 as a prognostic biomarker in the diagnosis of clear cell renal cell carcinoma (ccRCC). The identification of circSP3 as an encoding entity of a novel circular RNA enriches our understanding of the molecular underpinnings of ccRCC. This study paves the way for enhanced prognostic assessments and the formulation of targeted therapeutic approaches for patients afflicted with ccRCC.

## Supplementary Material

Supplementary figures and tables.

## Figures and Tables

**Table 1:** Association between clinicopathological variables and circSP3 expression in patients with ccRCC

Characteristics	Cases	CircSP3 expression		P-value
		Low	High	
Age (years)				
<60	49	27	22	0.0225*
≥60	51	23	28	
Gender				
Male	68	35	33	0.668
Female	32	15	17	
Tumor size				
≤5cm	42	24	18	0.224
>5cm	58	26	32	
AJCC stage_T				
T1/T2	61	36	25	0.0241*
T3/T4	39	14	25	
AJCC stage_N				
N0	98	48	50	0.153
N1	2	2	0	
AJCC stage_M				
M1	4	2	2	0.6098
MX	96	48	48	
GRADE				
I/II	72	40	32	0.0748
III/IV	28	10	18	
