# Supplementary material for: CircSP3 encodes SP3-461aa to promote ccRCC progression via stabilizing MYH9 and activating the PI3K-Akt signaling pathway
Source: J Cancer. 2024 Sep 16;15(18):5876–96. doi: 10.7150/jca.100706 (PMC11493002; doi:10.7150/jca.100706)
Supplement: Supplementary file 1 — Supplementary figures and tables. [file jcav15p5876s1.pdf]

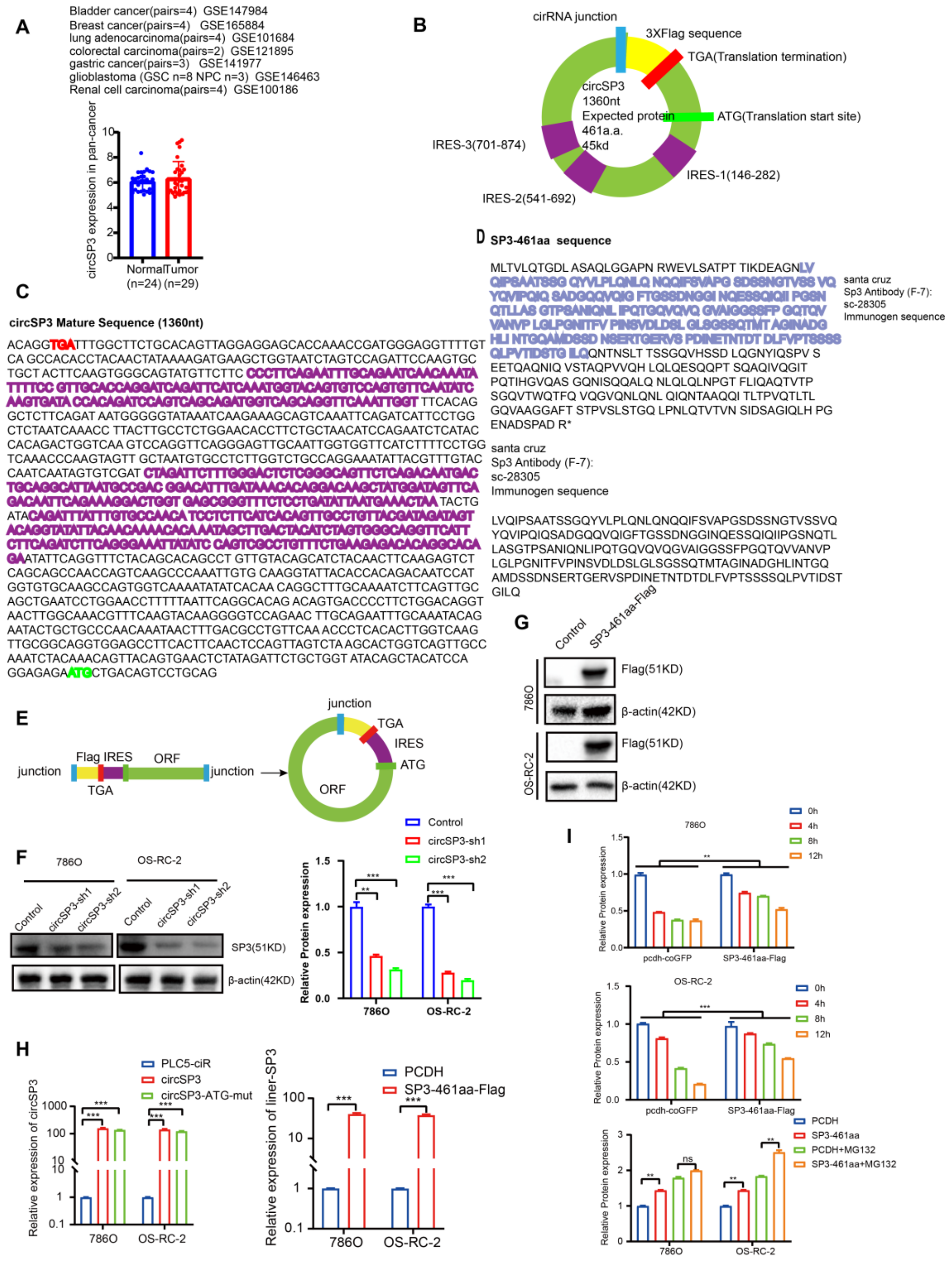

FigureS1. A. RT-qPCR was employed to conduct an expression analysis of circRNA hsa_circ_00233 in pan-cancer studies. **B.** The provided schematic visualization depicts the circSP3-FLAG construct, including the three IRES regions. **C.** CircSP3 mature sequence, the purple sequence is the predicted IRES sequence. **D.** The SP3-461aa sequence and the

Santacruz antibody sc-28305 immunogenic sequence. **E.** Schematic diagram visualizing the circSP3-FLAG construct, designed to facilitate identification of circSP3-encoded peptides. The FLAG tag sequence was precisely located in close proximity to the termination codon of the ORF. **F.** The detection of SP3-461aa proteins was achieved through the utilization of the Santa Cruz antibody sc-28305. **G.** The SP3-461aa-FLAG proteins were detected using FLAG antibodies. **H.** The cirSP3 and SP3 expression levels in ccRCC cells stably transfected with pLC5-ciR, cirSP3-OV, circ-SP3-ATG-mut, PCDH or SP3-461aa-FLAG (n = 3). **I.** The Protein quantification after CHX and MG132 treatment. Values were expressed as mean ± SD. *P<0.05, **P<0.01, ***P<0.001.

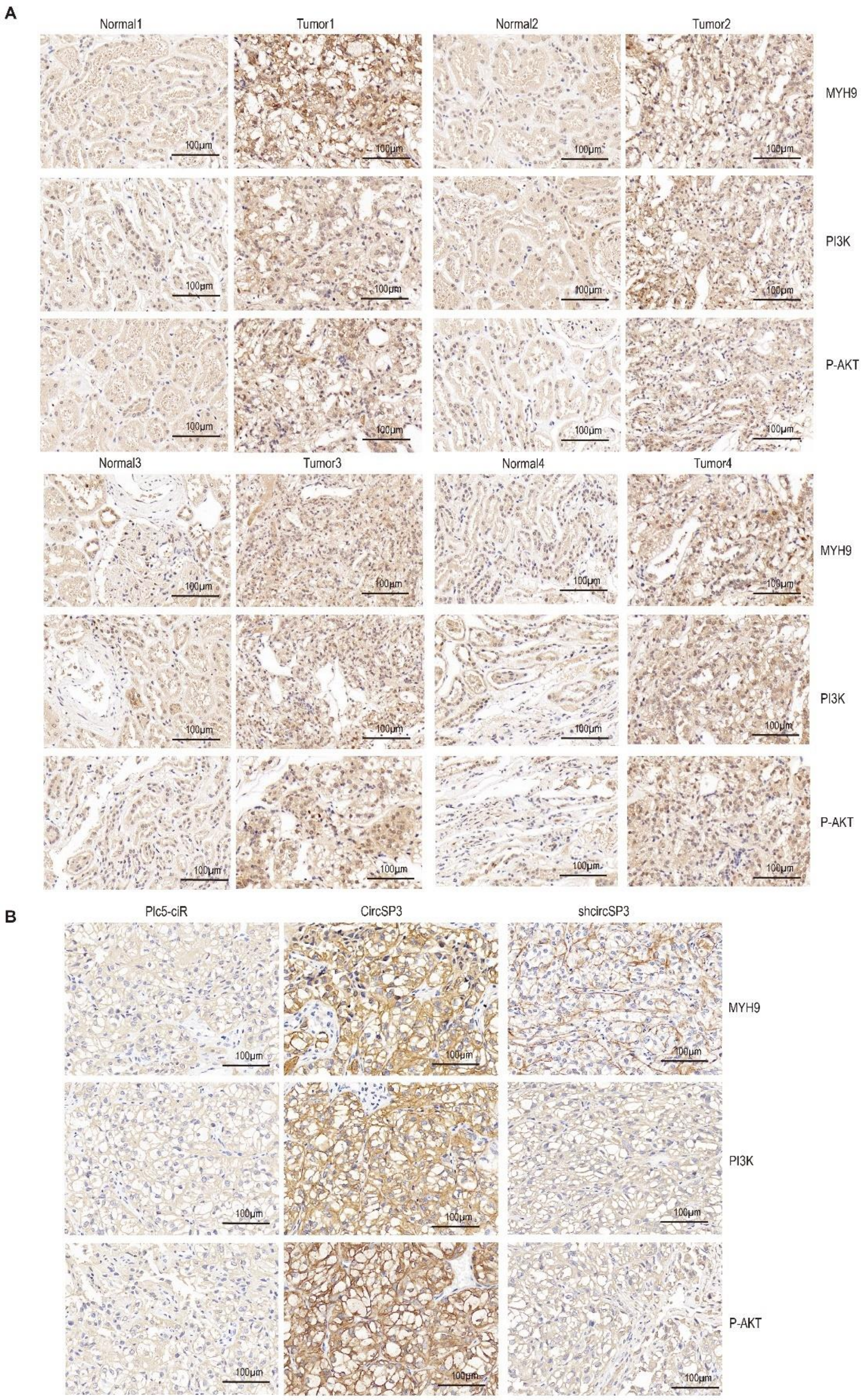

Figure S2. Expression of MYH9, PI3K, and AKT in tumor tissues. A. Immunohistochemical staining to detect the expression of MYH9, PI3K, and AKT in clear cell renal cell carcinoma tissues and adjacent normal tissues (n = 3). **B.** Immunohistochemical staining to detect the

expression of MYH9, PI3K, and AKT in xenografted samples (n = 3). Bars represent mean ± SD. *P<0.05, **P<0.01, ***P<0.001.

Table S2

Primer for PCR

Gene	Forward Primer	Reverse Primer
CircSP3/circSP3 Div	CACTGGTCAGTTGCCAAATC	GGTGCTCCTCCTAACTGTGC
CircSP3 Con	GCGACAGGTGATTTGGCTTCT	TACTGCCCACTTGAAGTAGCA
Liner-SP3-Flag	AATCAAACCTTACTTGCCTCTG	CCCGCTCACCAGTCCTTT
β-actin/β-actin Con	CACCATTGGCAATGAGCGGTTC	CACCATTGGCAATGAGCGGTTC
β-actin Div	CACCATTGGCAATGAGCGGTTC	CACCATTGGCAATGAGCGGTTC
GAPDH	GTCTCCTCTGACTTCAACAGCG	ACCACCCTGTTGCTGTAGCCAA
U6	CTCGCTTCGGCAGCACA	CTCGCTTCGGCAGCACA
SP3	CCTGAAGAGTGGCACAACTGTG	GCTCTGAGATGTGAGGTCTTCC
MYH9	ATCCTGGAGGACCAGAACTGCA	GGCGAGGCTCTTAGATTTCTCC

Primer for vector construction

Gene	Forward Primer	Reverse Primer
CircSP3-ATG-mut	TACATCCAGGAGAGAACGCTGACAGTCCTGCAG	CTGCAGGACTGTCAGCGTTCTCTCCTGGATGTA
SP3-461aa_flag	GATTACAAGGATGACGACGATAAGGACTATAAGGACGATGATGACAAGGACTACAAAGATGATGACGATAAATGAGCGGCCGCGAAGGATCT	CCTGTCTGCAGGACTGTCAGCATTCTCTCCTGGATGTAGCTGTATACC
CircSP3-sh1	GATCACAGTCCTGCAGACAGGTGATCTCGAGATCACCTGTCTGCAGGACTGTTTTTTG	GGCCCAAAAAACAGTCCTGCAGACAGGTGATCTCGAGATCACCTGTCTGCAGGACTGT
CircSP3-sh2	GATCAGTCCTGCAGACAGGTGATTTCTCGAGAAATCACCTGTCTGCAGGACTTTTTTG	GGCCCAAAAAAGTCCTGCAGACAGGTGATTTCTCGAGAAATCACCTGTCTGCAGGACT

Table S3.

Antibody

Vimentin	Proteintech
CCND1	Proteintech
ZEB1	Proteintech
PCNA	Proteintech
E-cadherin	Proteintech
N-cadherin	Proteintech

MYH9	Proteintech
VEGFA	Proteintech
PI3K	Proteintech
Phospho-AKT (Ser473)	Proteintech
β-actin	Proteintech
Gapdh	Proteintech
Ki-67	Proteintech
HA	Abcam
Flag	Abcam
Smyd2	Proteintech
